# Comparative evaluation of nutritional risk screening tools in hospitalized children with acute gastroenteritis

**DOI:** 10.3389/fnut.2026.1841518

**Published:** 2026-06-10

**Authors:** Yue Zhao, Ziyang Li, Mingyue Lin, Juan Zhang, Zhongxiang Li, Jiangyan Liu, Qianye Zhao, Kangwei Mao

**Affiliations:** 1 Department of Nursing, Lianyungang Maternity and Child Health Hospital, Lianyungang, Jiangsu, China; 2 Department of Pediatrics, The First People’s Hospital of Xuzhou, Xuzhou, Jiangsu, China; 3 Department of Pediatrics, The Affiliated Lianyungang Hospital of Xuzhou Medical University (The First People’s Hospital of Lianyungang), Lianyungang, Jiangsu, China; 4 Department of Pediatrics, Ganyu District Hospital of Traditional Chinese Medicine, Lianyungang, Jiangsu, China; 5 Department of Pediatrics, Lianyungang Maternity and Child Health Hospital, Lianyungang, Jiangsu, China

**Keywords:** acute gastroenteritis, malnutrition, nutritional risk screening, pediatric nutrition, PYMS, STAMP, STRONGkids

## Abstract

**Background:**

Malnutrition is common in hospitalized children with acute gastroenteritis, yet the optimal nutritional risk screening tool for this population remains unclear. This study aimed to compare the diagnostic performance and clinical utility of STRONGkids, PYMS, and STAMP in identifying malnutrition among hospitalized children with acute gastroenteritis.

**Methods:**

In this multicenter retrospective study, children hospitalized with acute gastroenteritis between January 2024 and January 2026 were identified from electronic medical records at admission. After exclusions, 855 children were included in the final analysis. Nutritional risk was retrospectively assessed using STRONGkids, PYMS, and STAMP. Nutritional status was classified according to body mass index-for-age z scores based on the World Health Organization growth reference. Receiver operating characteristic curve analysis was used to evaluate discriminatory performance, with pairwise comparisons performed using the DeLong test. Optimal cut-off values were determined using the Youden index, and decision curve analysis was performed to assess clinical utility.

**Results:**

Of the 855 children included, 82 were malnourished and 773 had normal nutritional status. Compared with children with normal nutritional status, malnourished children were younger and had more vomiting and diarrhea episodes, longer hospital stay, higher screening scores, and more severe dehydration. PYMS showed the best discriminatory performance, with an AUC of 0.860 (95% CI, 0.824–0.896), followed by STAMP at 0.796 (95% CI, 0.748–0.845) and STRONGkids at 0.717 (95% CI, 0.658–0.776). PYMS performed significantly better than both STRONGkids and STAMP, and STAMP also outperformed STRONGkids. At the optimal cut-off of 3, PYMS had the highest sensitivity, specificity, Youden index, and negative predictive value. Decision curve analysis also supported the superior clinical utility of PYMS.

**Conclusion:**

Among the three screening tools, PYMS demonstrated the best overall performance for identifying malnutrition in hospitalized children with acute gastroenteritis and may be the most suitable tool for early nutritional risk screening in this population.

## 1 Introduction

Acute gastroenteritis is one of the most common causes of hospitalization in children worldwide and remains an important contributor to pediatric morbidity (1). Although most cases are self-limiting, hospitalized children often present with vomiting, diarrhea, reduced oral intake, and varying degrees of dehydration (2, 3). These clinical manifestations may adversely affect nutritional status (4). Recent evidence has also highlighted the close relationship between acute diarrhea, clinical manifestations, and pediatric malnutrition, supporting the need to consider nutritional status in children with diarrheal illness (5). Malnutrition in hospitalized children has been associated with prolonged hospital stay, delayed recovery, increased risk of complications, and greater healthcare burden (6, 7). Therefore, early identification of children at nutritional risk is essential for timely nutritional assessment and intervention. However, direct nutritional assessment of all hospitalized children is often impractical in routine clinical settings. For this reason, although anthropometric indicators such as BMI-for-age z scores can also be used to assess nutritional status, rapid nutritional risk screening tools are increasingly recommended in routine clinical practice to identify children who may require timely intervention (8–10).

Several pediatric nutritional risk screening tools have been developed for hospital use, among which the Screening Tool for Risk on Nutritional Status and Growth (STRONGkids) (11), the Pediatric Yorkhill Malnutrition Score (PYMS) (12), and the Screening Tool for the Assessment of Malnutrition in Pediatrics (STAMP) (13) are among the most widely used. These tools differ in structure, scoring emphasis, and the clinical domains they assess (14, 15). STRONGkids is simple and easy to apply, PYMS places greater emphasis on anthropometric and recent nutritional changes, and STAMP incorporates both clinical diagnosis and nutritional intake. Previous studies have shown that these tools may perform differently across pediatric populations and clinical contexts, suggesting that their diagnostic value may not be equivalent (16–18). For children with acute gastroenteritis, reduced intake, gastrointestinal losses, and acute inflammatory stress often coexist, which may increase the risk of malnutrition (19). However, evidence directly comparing these tools in hospitalized children with acute gastroenteritis remains limited.

Therefore, this multicenter retrospective study aimed to compare the diagnostic performance of STRONGkids, PYMS, and STAMP for identifying malnutrition in hospitalized children with acute gastroenteritis. By clarifying their relative performance in this population, this study may contribute to the existing evidence regarding the applicability of pediatric nutritional risk screening tools in hospitalized children with acute gastroenteritis.

## 2 Materials and methods

### 2.1 Study design

This was a multicenter retrospective study conducted at three teaching hospitals in China. The study was designed to compare the performance of three pediatric nutritional risk screening tools, namely STRONGkids, PYMS, and STAMP, in hospitalized children with acute gastroenteritis. Relevant demographic, clinical, laboratory, and nutritional data were retrospectively extracted from the electronic medical records for analysis. The primary objective of the study was to evaluate and compare the discriminatory ability and clinical utility of these three screening tools for identifying malnutrition in this population.

### 2.2 Study population

Children hospitalized between January 2024 and January 2026 were retrospectively screened for eligibility using the corresponding ICD-10 diagnostic codes for acute gastroenteritis recorded in the electronic medical records at admission. A total of 1,182 children were initially assessed. Patients younger than 2 years were excluded because this study used BMI-for-age z scores as the primary anthropometric reference standard. Patients were also excluded if they had secondary gastrointestinal disorders or chronic systemic diseases, had received long-term corticosteroid or immunosuppressive therapy, or had incomplete anthropometric, laboratory, or screening tool data. After exclusion of 327 patients, 855 children were included in the final analysis.

**Figure 1:**
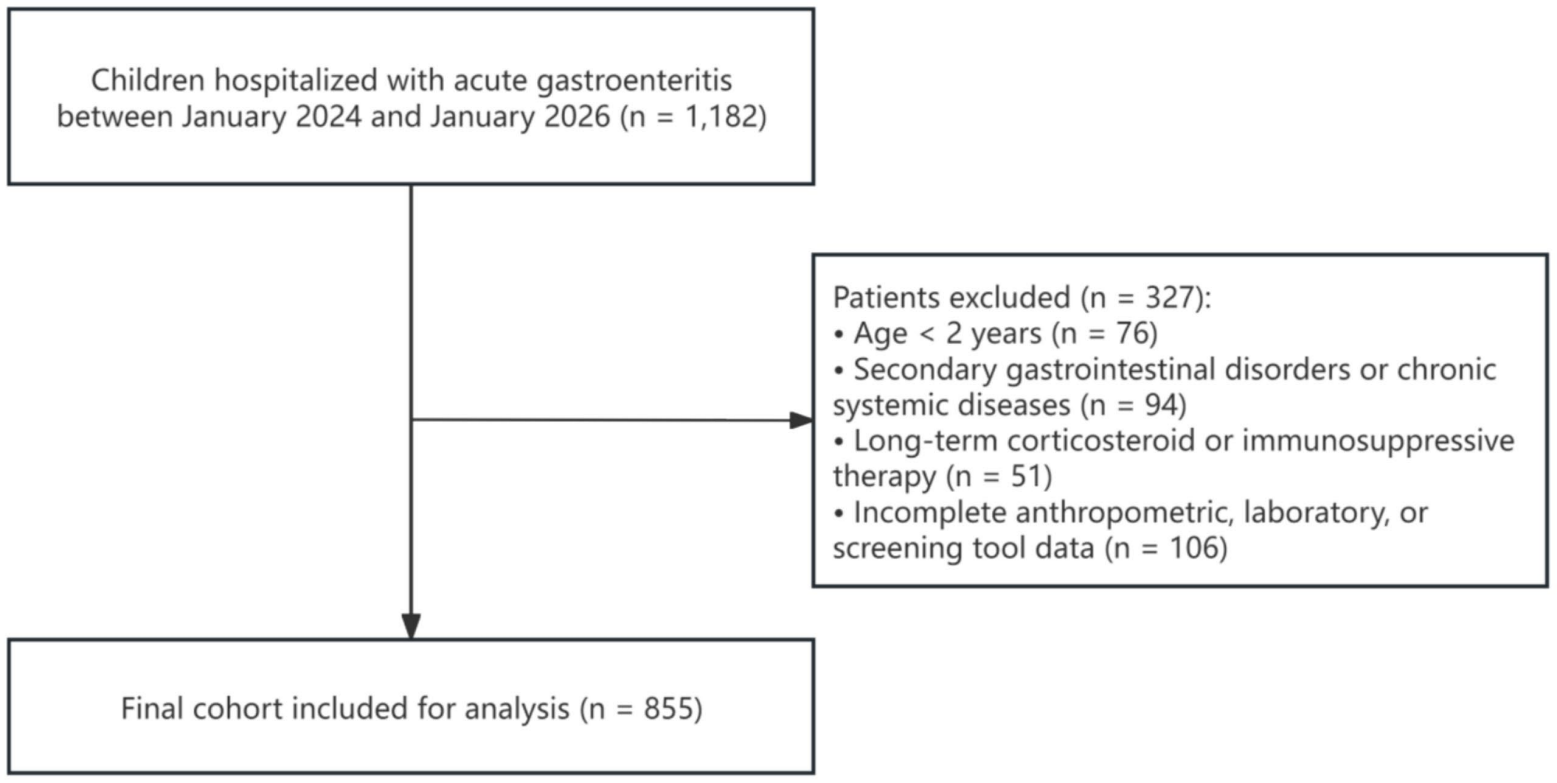
Flowchart of study population selection.

Among the excluded patients, 76 were excluded because they were younger than 2 years, 94 because of secondary gastrointestinal disorders or chronic systemic diseases, 51 because of long-term corticosteroid or immunosuppressive therapy, and 106 because of incomplete anthropometric, laboratory, or screening tool data. A flowchart of participant selection is shown in Figure 1.

### 2.3 Nutritional risk screening tools

Three pediatric nutritional risk screening tools, STRONGkids, PYMS, and STAMP, were evaluated in this study. For each participant, the three scores were retrospectively assessed according to the original scoring criteria using the information available in the electronic medical records. When required, anthropometric and clinical information documented at admission was used for retrospective scoring. To improve reliability, all scores were independently assessed by two trained investigators using a standardized scoring form. Any discrepancies were resolved through discussion, and if consensus could not be reached, a senior investigator made the final decision. Inter-rater reliability before consensus discussion was excellent, with intraclass correlation coefficients of 0.91 for STRONGkids, 0.94 for PYMS, and 0.92 for STAMP.

The three screening tools were evaluated against the predefined anthropometric reference standard for malnutrition used in this study. For statistical analysis, the scores were analyzed both as continuous variables and as dichotomized screening results according to the optimal cut-off values identified by receiver operating characteristic analysis.

### 2.4 Data collection and nutritional status classification

Clinical data were retrospectively extracted from the electronic medical record systems of the participating hospitals using a standardized data collection form. The collected variables included demographic characteristics, clinical manifestations, laboratory findings, hospitalization information, anthropometric measurements, and nutritional screening data. Specifically, the variables analyzed in this study included age, sex, C-reactive protein level, vomiting episodes, diarrhea episodes, length of hospital stay, dehydration severity, fever status, pathogen type, body weight, height, body mass index, and the STRONGkids, PYMS, and STAMP scores.

To improve consistency, prespecified definitions were applied during data extraction. Age was recorded at admission. Length of hospital stay was calculated as the number of days from admission to discharge. Vomiting and diarrhea episodes were extracted from admission records and inpatient documentation. These variables reflected the clinical severity of acute gastroenteritis but were not used to estimate quantitative fluid loss, because exact stool volume and cumulative fluid balance were not consistently available in the retrospective records. Dehydration severity, categorized as none, mild, moderate, or severe according to the clinical assessment documented in the medical records, was therefore used as a clinical indicator related to fluid loss. Fever status was defined according to the presence or absence of fever during the disease course. Pathogen type was classified as rotavirus, norovirus, bacterial, or unknown according to microbiological test results and discharge records.

Nutritional status was classified using anthropometric measurements at admission as the reference standard in this study. Body mass index was calculated as weight in kilograms divided by height in meters squared, and BMI-for-age z scores were determined according to the World Health Organization growth reference. Because all included children were aged 2 years or older, BMI-for-age was used as the primary anthropometric indicator for nutritional classification. Children with a BMI-for-age z score below −2 standard deviations were classified as malnourished, whereas those with a z score of −2 standard deviations or higher were classified as having normal nutritional status. Because anthropometric measurements were obtained at admission, dehydration-related changes in body weight could not be completely excluded. Dehydration severity was therefore recorded as an important clinical variable and compared between nutritional status groups, but BMI-for-age z scores were not corrected for dehydration because pre-illness or post-rehydration weight was not consistently available.

### 2.5 Sample size calculation

Sample size was estimated using receiver operating characteristic curve analysis, the primary analytical approach of this study. Assuming a two-sided *α* of 0.05, a power of 80%, an anticipated area under the curve of 0.80, a minimum acceptable area under the curve of 0.70, and an approximate malnutrition to non-malnutrition ratio of 1:9, the minimum required sample size was estimated to be 604 children, including at least 59 malnourished and 545 non-malnourished participants. Therefore, the final sample of 855 children was considered sufficient for the planned diagnostic performance analyses. The sample size was estimated for the primary ROC analysis rather than for subgroup-specific comparisons, and the relatively low prevalence of malnutrition in this cohort may have limited the statistical power for subgroup analyses.

### 2.6 Statistical analysis

Statistical analyses were performed to compare baseline characteristics between children with normal nutritional status and those with malnutrition, and to evaluate the screening performance of STRONGkids, PYMS, and STAMP. Continuous variables are presented as median with interquartile range and were compared using the Mann–Whitney U test. Categorical variables are presented as number and percentage and were compared using the chi-square test or Fisher’s exact test, as appropriate. Receiver operating characteristic curve analysis was used to assess the discriminatory ability of STRONGkids, PYMS, and STAMP for identifying malnutrition. The area under the curve and its 95% confidence interval were calculated for each tool. Pairwise comparisons of the area under the curve were performed using the DeLong test. The optimal cut-off value for each screening tool was determined using the Youden index. Sensitivity, specificity, positive predictive value, and negative predictive value were calculated to evaluate diagnostic performance at the optimal threshold. Confidence intervals for sensitivity and specificity at the fixed optimal cut-off values were calculated using the exact binomial method. Decision curve analysis was performed to compare the clinical utility of the three screening tools across a range of threshold probabilities. A two-sided *p* value of less than 0.05 was considered statistically significant.

## 3 Results

### 3.1 Baseline characteristics

A total of 855 hospitalized children with acute gastroenteritis were included in the final analysis, including 773 children with normal nutritional status and 82 classified as malnourished. Compared with children with normal nutritional status, those in the malnourished group were younger and had more vomiting episodes, more diarrhea episodes, and a longer hospital stay. Diarrhea frequency was analyzed as a clinical manifestation of acute gastroenteritis. However, the exact duration of diarrhea before admission was not consistently documented across centers and therefore was not included in the formal analysis. They also had significantly higher STRONGkids, PYMS, and STAMP scores. Severe dehydration was more frequent among malnourished children. No significant differences were observed in CRP level, sex, fever, or pathogen type between the two groups (Table 1).

**Table 1:** Baseline characteristics of the study population.

Variable	Normal (*n* = 773)	Malnourished (*n* = 82)	*p*-value
Age, years (median [IQR])	9.00 [6.00, 13.00]	8.00 [4.00, 11.00]	0.011
CRP, mg/L (median [IQR])	4.30 [2.50, 7.30]	3.90 [2.50, 7.88]	0.769
Vomiting episodes (median [IQR])	2.00 [1.00, 3.00]	2.00 [1.00, 4.00]	0.031
Diarrhea episodes (median [IQR])	4.00 [2.00, 5.00]	5.00 [3.00, 7.00]	0.004
Length of stay, days (median [IQR])	4.00 [3.00, 5.00]	4.00 [3.00, 6.00]	0.039
STRONGkids score (median [IQR])	1.00 [0.00, 2.00]	2.00 [1.00, 3.00]	<0.001
PYMS score (median [IQR])	1.00 [1.00, 3.00]	4.00 [3.00, 5.00]	<0.001
STAMP score (median [IQR])	2.00 [1.00, 3.00]	4.00 [3.00, 5.00]	<0.001
Male, *n* (%)	397 (51.4)	50 (61.0)	0.104
Dehydration severity, *n* (%)			0.003
None	353 (45.7)	26 (31.7)	
Mild	253 (32.7)	28 (34.1)	
Moderate	99 (12.8)	10 (12.2)	
Severe	68 (8.8)	18 (22.0)	
Fever, *n* (%)	456 (59.0)	44 (53.7)	0.349
Pathogen type, *n* (%)			0.838
Rotavirus	120 (15.5)	13 (15.9)	
Norovirus	190 (24.6)	17 (20.7)	
Bacterial	282 (36.5)	30 (36.6)	
Unknown	181 (23.4)	22 (26.8)	

### 3.2 Comparative discriminatory performance of the three screening tools

**Figure 2:**
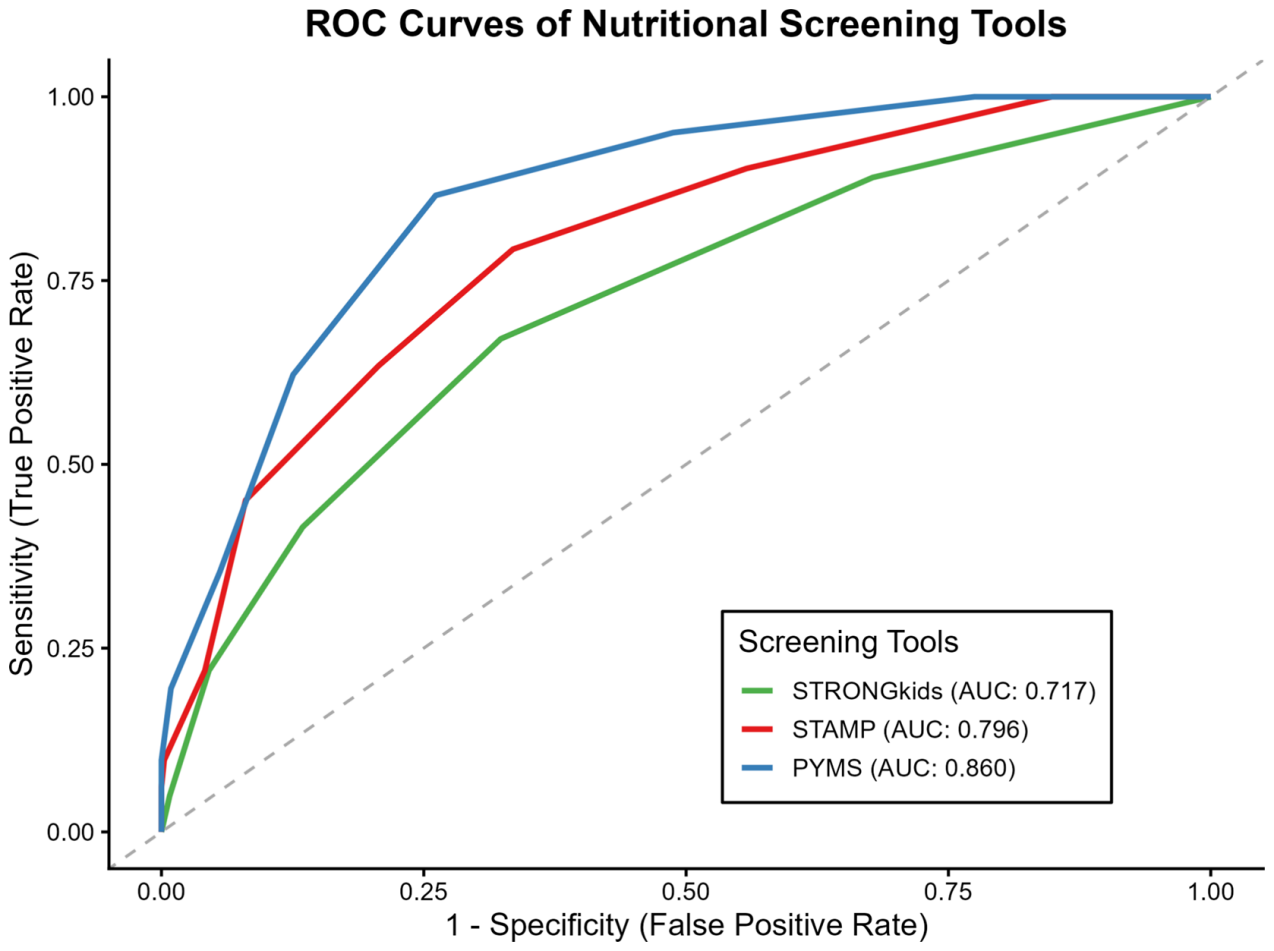
Comparative ROC analysis of STRONGkids, PYMS, and STAMP for the discrimination of malnutrition.

Receiver operating characteristic curve analysis was performed to evaluate the discriminatory performance of STRONGkids, PYMS, and STAMP for identifying malnutrition (Figure 2). PYMS showed the best discriminatory performance, with an AUC of 0.860 (95% CI, 0.824–0.896), followed by STAMP at 0.796 (95% CI, 0.748–0.845) and STRONGkids at 0.717 (95% CI, 0.658–0.776). DeLong test showed that PYMS performed significantly better than STRONGkids (ΔAUC = 0.143, Z = 4.014, *p* < 0.001) and STAMP (ΔAUC = 0.064, Z = 2.158, *p* = 0.031), while STAMP also significantly outperformed STRONGkids (ΔAUC = 0.079, Z = 2.103, *p* = 0.035) (Table 2).

**Table 2:** Pairwise comparison of ROC curves among STRONGkids, PYMS, and STAMP using DeLong test.

Comparison	AUC 1 (95% CI)	AUC 2 (95% CI)	ΔAUC	Z	p-value	95% CI for ΔAUC
PYMS vs. STRONGkids	0.860 (0.824–0.896)	0.717 (0.658–0.776)	0.143	4.014	<0.001	0.073–0.213
STAMP vs. STRONGkids	0.796 (0.748–0.845)	0.717 (0.658–0.776)	0.079	2.103	0.035	0.005–0.153
PYMS vs. STAMP	0.860 (0.824–0.896)	0.796 (0.748–0.845)	0.064	2.158	0.031	0.006–0.122

### 3.3 Diagnostic performance at optimal cut-off values

The diagnostic performance of the three nutritional risk screening tools at their optimal cut-off values, as determined by the Youden index, is presented in Table 3. The optimal cut-off value was 2 for STRONGkids and 3 for both STAMP and PYMS. PYMS showed the highest sensitivity, specificity, Youden index, positive predictive value, and negative predictive value among the three tools. STAMP showed intermediate performance, whereas STRONGkids had the lowest Youden index. The high negative predictive value of PYMS suggests that it may be useful for ruling out malnutrition in this population, although this finding should be interpreted in relation to the BMI-for-age z score reference standard.

**Table 3:** Diagnostic performance of STRONGkids, PYMS, and STAMP at optimal cut-off values determined by the Youden index.

Tool	Optimal cut-off	Sensitivity (95% CI)	Specificity (95% CI)	Youden index	PPV (%)	NPV (%)
STRONGkids	2	0.671 (0.558–0.771)	0.677 (0.642–0.709)	0.347	18.033	95.091
STAMP	3	0.793 (0.689–0.874)	0.665 (0.630–0.698)	0.458	20.062	96.798
PYMS	3	0.866 (0.773–0.931)	0.739 (0.706–0.769)	0.605	26.007	98.110

PPV, positive predictive value; NPV, negative predictive value.

### 3.4 Score distribution and clinical utility

**Figure 3:**
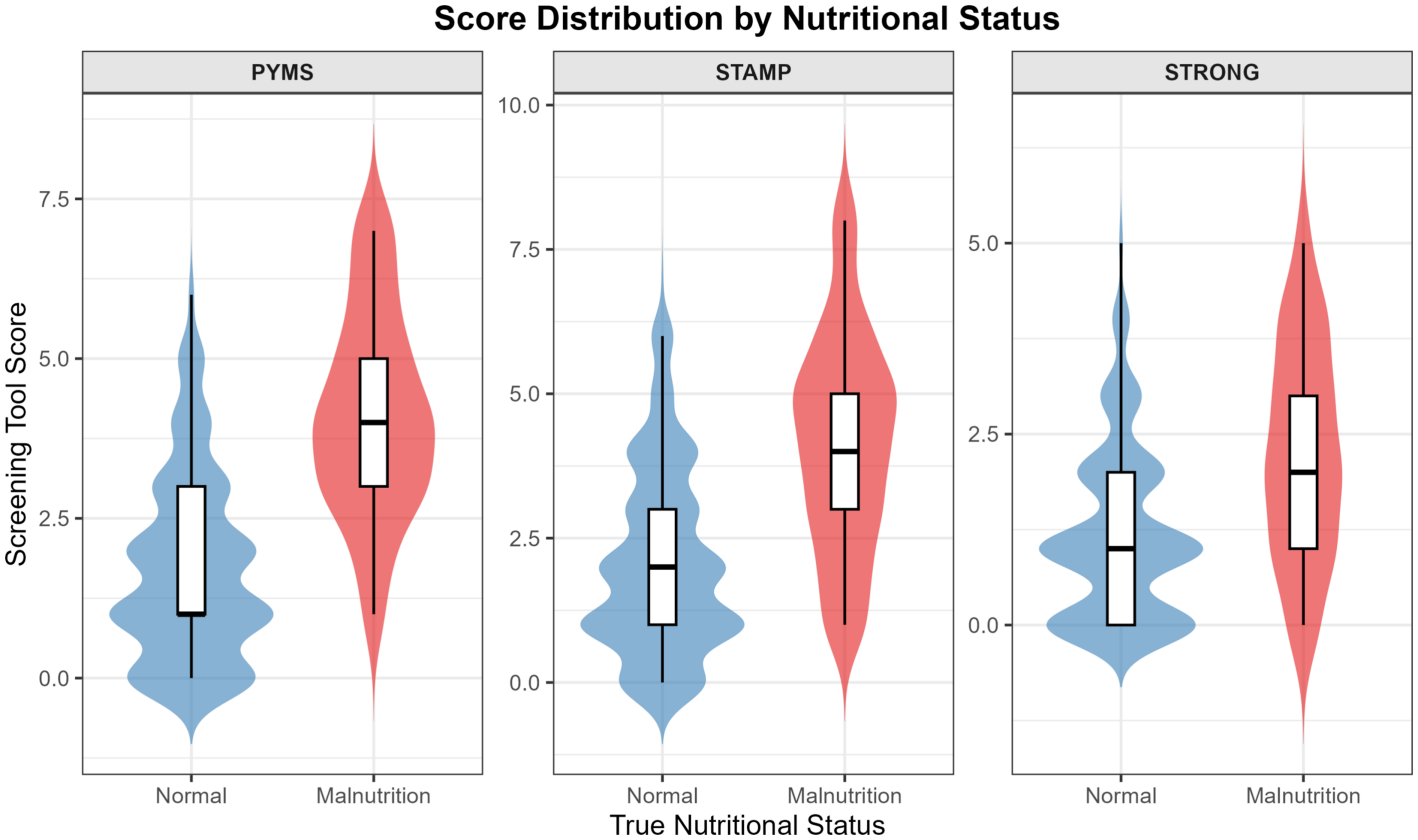
Violin plots illustrating score distribution of STRONGkids, PYMS, and STAMP according to true nutritional status.

**Figure 4:**
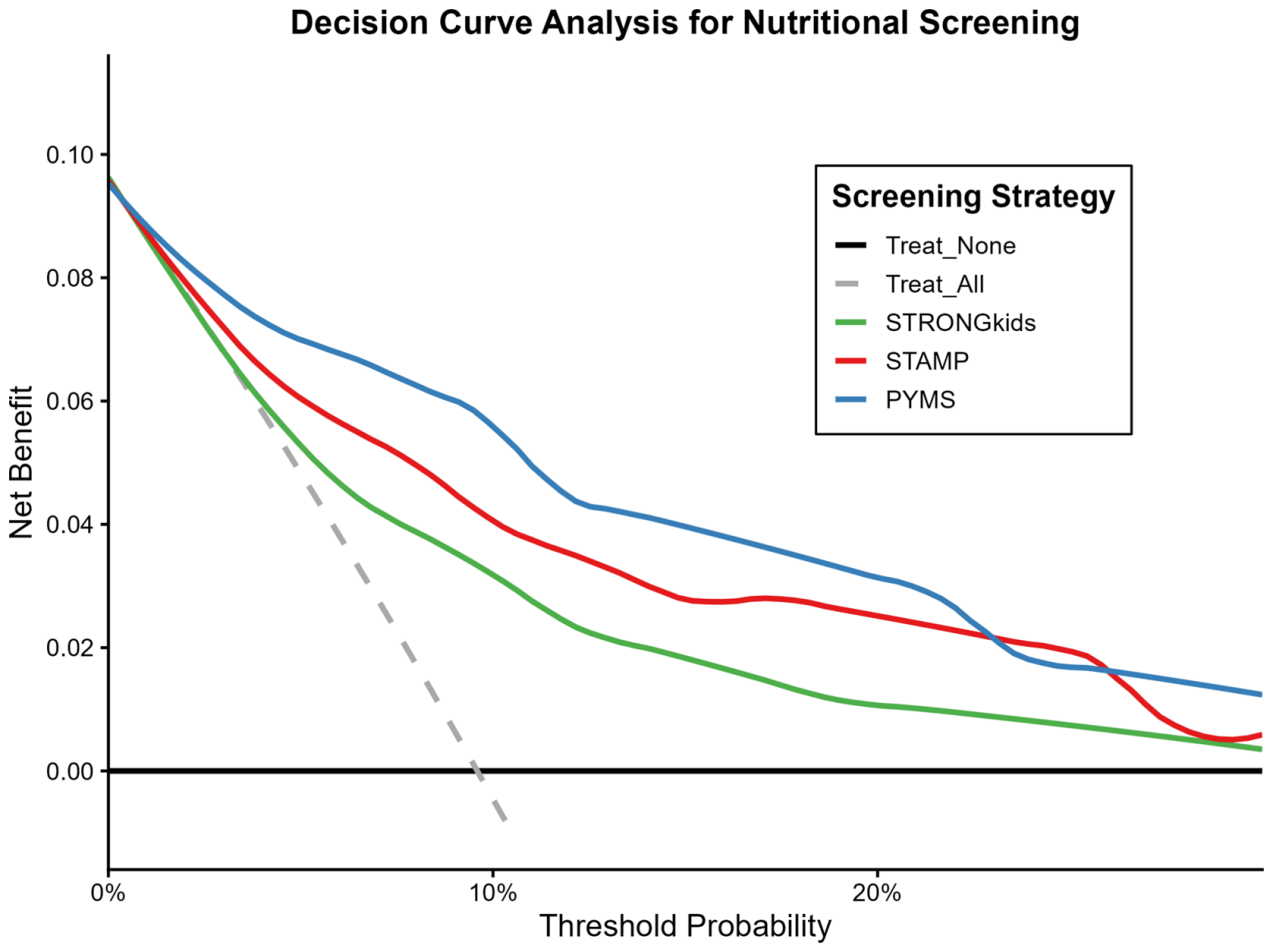
Decision curve analysis demonstrating the clinical utility of STRONGkids, PYMS, and STAMP for malnutrition screening.

The distributions of STRONGkids, PYMS, and STAMP scores according to true nutritional status are shown in Figure 3. For all three screening tools, score distributions were shifted toward higher values in malnourished children compared with children with normal nutritional status. Among them, PYMS showed the clearest separation between the two groups, which was in line with its superior discriminatory performance in the ROC analysis. As shown in Figure 4, decision curve analysis further demonstrated the clinical utility of the three screening tools across a range of threshold probabilities. PYMS yielded the highest net benefit across most of the clinically relevant threshold range, followed by STAMP, whereas STRONGkids showed the lowest net benefit overall.

## 4 Discussion

In this multicenter retrospective study, we compared the performance of three commonly used pediatric nutritional risk screening tools, namely STRONGkids, PYMS, and STAMP, for identifying malnutrition in hospitalized children with acute gastroenteritis. The main finding was that PYMS demonstrated the best overall performance among the three tools. It showed the highest discriminatory ability, the best diagnostic performance at the optimal cut-off value, and the greatest clinical utility on decision curve analysis. By comparison, STRONGkids showed the lowest discriminatory performance, whereas STAMP showed intermediate performance. These results indicate that PYMS may be the most suitable screening tool for identifying malnutrition in hospitalized children with acute gastroenteritis.

Malnutrition is an important but often underrecognized problem in children admitted with acute gastroenteritis (20, 21). Acute gastrointestinal symptoms such as vomiting, diarrhea, poor oral intake, and dehydration can rapidly compromise nutritional status, particularly in young children with limited physiological reserves (22). In the present study, children classified as malnourished were younger and had more frequent vomiting and diarrhea episodes, longer hospital stay, and more severe dehydration than those with normal nutritional status. These findings are clinically plausible and support the validity of the nutritional classification used in this study. They also underscore the close interaction between acute disease burden and nutritional vulnerability in this population (23).

Among the three tools evaluated, PYMS achieved the highest AUC and significantly outperformed both STRONGkids and STAMP. This superior performance may be related to the design characteristics of PYMS. Compared with the other two tools, PYMS places greater emphasis on anthropometric status and recent nutritional changes, which are likely to be closely associated with the anthropometric reference standard used in this study (24). Because malnutrition was defined according to BMI-for-age z scores based on the World Health Organization growth reference, a screening tool that more directly reflects body size and recent nutritional decline would be expected to show better agreement with the reference standard (25). This may partly explain why PYMS showed clearer separation between children with normal nutritional status and those with malnutrition.

Another possible explanation is that PYMS may be better suited to the clinical profile of children hospitalized with acute gastroenteritis. In this setting, nutritional risk is often driven by recent reductions in intake and rapid nutritional deterioration related to acute illness rather than by complex chronic comorbidity (26). PYMS is structured to capture such short-term nutritional risk through its assessment of anthropometric status, recent weight-related concerns, and the likely nutritional impact of the presenting illness. By contrast, STRONGkids includes a broader subjective clinical component, which may be easier to apply but may also reduce specificity in a population in whom acute symptoms are common. STAMP incorporates diagnosis-related risk and nutritional intake, but its overall performance in our cohort remained lower than that of PYMS.

The relatively lower performance of STRONGkids in this study deserves attention. STRONGkids is widely used because of its simplicity and practicality in routine clinical care (10, 27). However, its stronger reliance on subjective clinical judgment and disease-related risk may limit its ability to distinguish between transient acute illness and true nutritional impairment in children with acute gastroenteritis. Since many hospitalized children with acute gastroenteritis share common symptoms, STRONGkids may overclassify children as at risk and fail to distinguish true malnutrition from transient illness-related changes. This may have contributed to its lower specificity and lower overall AUC.

STAMP showed moderate performance, ranking between PYMS and STRONGkids. This finding suggests that STAMP may still have value as a nutritional risk screening tool in this population, but its discriminatory ability appears less robust than that of PYMS. One possible reason is that although STAMP includes anthropometric assessment, it also relies on diagnosis-related weighting that may not fully capture the variation in nutritional vulnerability among children hospitalized for the same broad condition. In acute gastroenteritis, disease severity and nutritional consequences may differ considerably even within the same diagnostic category, which may reduce the discriminative contribution of diagnosis-based scoring alone.

Although BMI-for-age z scores can be calculated at admission and provide an anthropometric reference for nutritional classification, nutritional risk screening tools remain clinically important because they allow rapid bedside identification of children who may be at increased risk of nutritional deterioration and require further assessment or early intervention (28). Early identification of malnutrition or high nutritional risk is essential for timely referral, nutritional assessment, and intervention (29, 30). In children hospitalized with acute gastroenteritis, an effective screening tool may help clinicians recognize vulnerable patients early in the admission process and prioritize nutritional support for those most likely to benefit. Based on our results, PYMS may be the most suitable option among the three evaluated tools for routine nutritional risk screening in this setting. Its relatively high sensitivity and negative predictive value also suggest that it may be particularly useful for ruling out malnutrition in children at low risk, thereby helping to optimize resource allocation in busy pediatric wards.

This study also has several strengths. First, this study focused on hospitalized children with acute gastroenteritis, a clinically common population for which comparative evidence on pediatric nutritional risk screening tools remains limited. Second, the study directly compared three widely used screening tools within the same cohort, allowing a comparative evaluation of their performance. Third, beyond standard ROC analysis, we used decision curve analysis to assess clinical utility, which provides additional information regarding the potential net benefit of each tool in real-world practice.

Several limitations should be acknowledged. First, this was a retrospective study, and the three screening scores were reconstructed from electronic medical records. Although standardized forms and predefined criteria were used, incomplete or inconsistent documentation may still have affected scoring accuracy. Pre-admission diarrhea duration, exact stool volume, and cumulative fluid balance were not consistently recorded, which limited further evaluation of the relationship between illness duration, fluid loss, and nutritional status at admission. Future prospective studies should collect diarrhea duration, fluid loss indicators, and post-rehydration anthropometric data in a standardized manner. Second, BMI-for-age z scores were used as the reference standard for malnutrition. This indicator is widely accepted, but it may not fully capture all aspects of pediatric malnutrition. Because body weight was measured at admission, dehydration may have influenced BMI-for-age z scores and caused misclassification in some children. Reliable correction using pre-illness or post-rehydration weight was not possible in this retrospective study. In addition, because PYMS includes items more closely aligned with BMI-for-age z scores than STRONGkids or STAMP, its superior performance may have been partly influenced by incorporation bias. Therefore, our findings indicate better agreement with the selected reference standard rather than definitive superiority in all clinical settings. Third, the study population was limited to hospitalized children with acute gastroenteritis from participating centers, which may limit the generalizability of the findings.

Future prospective studies are warranted to validate these findings in broader pediatric populations and to further examine the applicability of PYMS in routine clinical workflows. In addition, future research should explore whether improved screening translates into earlier nutritional intervention and better clinical outcomes.

## 5 Conclusion

In conclusion, PYMS demonstrated the best overall performance among the three pediatric nutritional risk screening tools for identifying malnutrition in hospitalized children with acute gastroenteritis. It may therefore be the most suitable option for early nutritional risk screening in this population. Further prospective studies are warranted to validate these findings and to determine whether its use improves nutritional management and clinical outcomes.

## Data Availability

The raw data supporting the conclusions of this article will be made available by the authors, without undue reservation.
